# Limited-edition smokeless tobacco packaging: Behind the camouflage

**DOI:** 10.18332/tid/110676

**Published:** 2019-08-02

**Authors:** Elizabeth T. Couch, Janelle Urata, Benjamin W. Chaffee

**Affiliations:** 1 Division of Oral Epidemiology and Dental Public Health, University of California, San Francisco, United States

**Keywords:** smokeless tobacco, packaging, camo cans, limited-edition packaging

**Dear Editor,**

Moist snuff, the top-selling smokeless tobacco (ST) product in the US, is particularly popular among young males participating in outdoor sports, such as rodeo and baseball^1,2^. Tobacco manufacturers have a long history of promoting their products with sports imagery and narratives related to individualism and alpha masculinity^3^. Under recent marketing efforts to target male outdoor enthusiasts, the two largest ST manufacturers, U.S. Smokeless Tobacco Company (parent: Altria Group, Inc.) and American Snuff Company (parent: Reynolds American Inc.), have released limited-edition camouflage (‘camo cans’) and fishing-themed packaging for their most popular moist snuff brands, ‘Copenhagen’ and ‘Grizzly’, respectively.

The U.S. Smokeless Tobacco Company first released ‘camo cans’ in 2009 to promote the launch of Copenhagen Long Cut Wintergreen moist snuff. In 2013, the American Snuff Company followed suit. Each year in the Fall, new limited-edition camo cans are released to coincide with deer hunting season (Figure 1a). Direct mailer and email announcements encourage consumers to visit product websites to redeem coupons, obtain promotional items, and play online ‘hunting’ games. Messages include dollar-off coupons with the text: ‘Hope you are wearing camo. Cause here comes a buck’.

**Figure 1:**
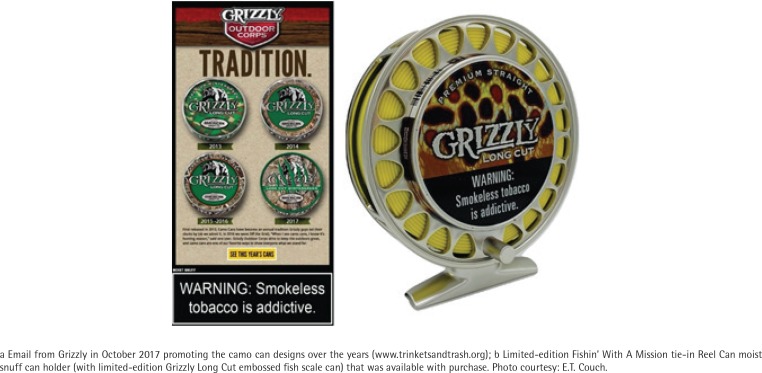
Grizzly Limited-Edition Camouflage- and Fish-Themed Smokeless Tobacco Packaging

The limited-edition packaging reinforces ST users as rugged sportsmen, reminiscent of decades-long tobacco marketing strategies featuring athlete endorsements^2,4^ and sporting event sponsorships^2^, now prohibited in the US. Under increased marketing restrictions, product packaging has gained importance in attracting users and maintaining market share^5,6^. For cigarettes, adult consumers rate limited-edition packaging as the most attractive and attention-grabbing^7^. Among adolescent males, seasonal ST promotions have been shown to stimulate interest and create urgency to purchase^8^, potentially adding perceived value to the product through rarity and exclusivity^9^.

Limited-edition packaging can link brands to specific identities and values. In Summer 2018, the American Snuff Company released a series of limited-edition fishing-themed cans to promote Grizzly’s ‘Fishin’ With a Mission’ corporate social responsibility (CSR) campaign that claimed a commitment to sportfish and habitat conservation. Campaign promotions also included a website link to enter to win prizes, redeem coupons, or find collectables, such as the Reel Can, a plastic novelty item used for prominent display of a moist snuff can (Figure 1b).

Tobacco manufacturers use CSR and philanthropy to improve their image and influence public health policy^10,11^, including environmental campaigns focused on planting trees, recycling cigarette butts, and manufacturing ‘earth friendly’ tobacco^11,12^. However, the same companies that produce and market ST are also the top manufacturers of cigarettes. While the environmental impact of ST is less studied, the harm to wildlife and habitats caused by cigarettes is real^13-15^.

Limited-edition ST packaging is used to communicate masculine stereotypes, introduce games and novelty items, promote CSR campaigns, and reinforce the relationship between ST and sport. Limited-edition packaging plausibly contributes to misconceptions about the environmental impact of tobacco production and increases youth appeal. Restrictions on advertising and promotion through online and direct mail may reduce the appeal and urgency to purchase limited-edition ST products. Moreover, mandatory plain packaging for all products, including ST, would eliminate the use of limited-edition packaging to communicate with the public, thereby reducing tobacco use in the US.

## CONFLICTS OF INTEREST

The authors have completed and submitted an ICMJE form for disclosure of potential conflicts of interest and they declare that they have no competing interests, financial or otherwise, related to the current work. All the authors report grants from US National Institutes of Health National Heart Lung and Blood Institute and Food and Drug Administration Center for Tobacco Products, during the conduct of the study.

## References

[1] Agaku IT, Singh T, Jones SE (2015). Combustible and smokeless tobacco use among high school athletes - United States, 2001-2013. MMWR Morb Mortal Wkly Rep.

[2] Ling PM, Haber LA, Wedl S (2010). Branding the rodeo: a case study of tobacco sports sponsorship. Am J Public Health.

[3] Dewhirst T, Sparks R (2003). Intertextuality, tobacco sponsorship of sports, and adolescent male smoking culture: A selective review of tobacco industry documents. J Sport Soc Issues.

[4] Severson HH, Klein K, Lichtensein E, Kaufman N, Orleans CT (2005). Smokeless tobacco use among professional baseball players: survey results, 1998 to 2003. Tob Control.

[5] Moodie C, Hastings G (2010). Tobacco packaging as promotion. Tob Control.

[6] Kotnowski K, Hammond D (2013). The impact of cigarette pack shape, size and opening: evidence from tobacco company documents. Addiction.

[7] Gallopel-Morvan K, Moodie C, Hammond D, Eker F, Beguinot E, Martinet Y (2012). Consumer perceptions of cigarette pack design in France: a comparison of regular, limited edition and plain packaging. Tob Control.

[8] Couch ET, Darius EF, Walsh MM, Chaffee BW (2017). ST product characteristics and relationships with perceptions and behaviors among rural adolescent males: a qualitative study. Health Educ Res.

[9] Ford A, Moodie C, Hastings G (2012). The role of packaging for consumer products: Understanding the move towards ‘plain’ tobacco packaging. Addict Res Theory.

[10] Tesler LE, Malone RE (2008). Corporate philanthropy, lobbying, and public health policy. Am J Public Health.

[11] Epperson AE, Henriksen L, Prochaska JJ (2017). Natural American Spirit brand marketing casts health halo around smoking. Am J Public Health.

[12] Gonzalez M, Ling PM, Glantz SA (2012). Planting trees without leaving home: tobacco company direct-to-consumer CSR efforts. Tob Control.

[13] Novotny TE, Hardin SN, Hovda LR, Novotny DJ, McLean MK, Khan S (2011). Tobacco and cigarette butt consumption in humans and animals. Tob Control.

[14] Slaughter E, Gersberg RM, Watanabe K, Rudolph J, Stransky C, Novotny TE (2011). Toxicity of cigarette butts, and their chemical components, to marine and freshwater fish. Tob Control.

[15] Zafeiridou M, Hopkinson NS, Voulvoulis N (2018). Cigarette smoking: an assessment of tobacco’s global environmental footprint across its entire supply chain. Environ Sci Technol.

